# Relationship Between Fatty Acid Binding Protein 4 and Liver Fat in Individuals at Increased Cardiometabolic Risk

**DOI:** 10.3389/fphys.2021.781789

**Published:** 2021-12-13

**Authors:** Ricardo Rodríguez-Calvo, Juan Moreno-Vedia, Josefa Girona, Daiana Ibarretxe, Neus Martínez-Micaelo, Jordi Merino, Nuria Plana, Lluis Masana

**Affiliations:** ^1^Vascular Medicine and Metabolism Unit, Research Unit on Lipids and Atherosclerosis, “Sant Joan” University Hospital, Universitat Rovira i Virgili, Pere Virgili Health Research Institute (IISPV), Reus, Spain; ^2^Spanish Biomedical Research Centre in Diabetes and Associated Metabolic Disorders (CIBERDEM), Institute of Health Carlos III, Madrid, Spain; ^3^Diabetes Unit and Center for Genomic Medicine, Massachusetts General Hospital, Boston, MA, United States; ^4^Program in Medical and Population Genetics, Broad Institute, Cambridge, MA, United States; ^5^Department of Medicine, Harvard Medical School, Boston, MA, United States

**Keywords:** FABP4 (fatty acid binding protein 4), FLI (fatty liver index), NAFLD (non-alcoholic fatty liver disease), metabolic patients, fatty liver

## Abstract

**Background:** Liver steatosis is considered the onset of the non-alcoholic fatty liver disease (NAFLD), a major public health challenge. Nevertheless, NAFLD detection and diagnosis remain a difficult task. Fatty acid binding protein 4 (FABP4) has been proposed as potential biomarker for the ectopic fat accumulation in non-adipose tissues, although its role reflecting liver steatosis in metabolic patients is not fully explored. The aim of this study was to assess the relationship between FABP4 and the fatty liver index (FLI) in metabolic patients and to evaluate its potential role in the fatty liver disease.

**Methods:** A cross-sectional study involving 389 participants at increased cardiometabolic risk was performed. FLI was calculated in order to assess liver fatty disease and a FLI ≥ 60 was considered to define liver steatosis. The serum FABP4 levels were assessed by using a sandwich enzyme-linked immunosorbent assay. Multivariable regression models were used to examine the associations of FABP4 with fatty liver after adjusting for demographic and clinical characteristics.

**Results:** Both, FLI and serum FABP4 levels were upregulated in diabetic, obese, and metabolic syndrome patients. Serum FABP4 levels were higher in individuals with liver steatosis. Serum FABP4 were robustly associated with FLI in metabolic patients in both linear and logistic regression analyses.

**Conclusion:** Our findings show that the serum FABP4 is associated to liver steatosis in metabolic patients.

## Introduction

Non-alcoholic fatty liver disease (NAFLD) is considered the main cause of chronic liver disease in the Western societies (Bonora and Targher, 2012; Wong et al., 2015; Kanwal et al., 2016). NAFLD is a progressive pathology, encompassing the so-called non-alcoholic fatty liver (NAFL), characterized by an excessive accumulation of triglycerides in the liver (Cohen et al., 2011). If no action is taken, NAFL can progress to non-alcoholic steatohepatitis (NASH), cirrhosis, and eventually hepatocellular carcinoma (Sun and Karin, 2012).

Despite its increased recognition as main cause of chronic liver disease, NAFLD remains largely underdiagnosed and undertreated, since effective methods for its diagnosis and monitoring are currently lacking. Most patients with NAFLD are asymptomatic, and the progression to advanced stages of the disease is often unnoticed. Liver biopsy is required for the definitive diagnosis of the most advanced stages of NAFLD (for review see Friedman et al., 2018), but has several limitations, such as invasiveness, potentially life-threatening complications or sampling variability, among others. Additionally, it is not suitable for the monitoring of the disease. Liver steatosis can be accurately quantified by magnetic resonance spectroscopy, but it requires specialized infrastructure that is not available in all hospitals. Therefore non-invasive biomarkers are needed for the identification and monitoring of the disease. Increasing evidences suggest that the amount of fat deposited in the liver can be estimated using non-invasive clinical tools available to all clinicians such as the fatty liver index (FLI) (Bedogni et al., 2006; Akinkugbe et al., 2017). FLI is an algorithm obtained from routinely measured clinical variables widely used in epidemiological studies (Koehler et al., 2013; Meffert et al., 2014) that has been highly correlated with the amount of liver fat measured by magnetic resonance spectroscopy (Cuthbertson et al., 2014).

We have recently reported that the circulating levels of the fatty acid binding protein 4 (FABP4), reflects the myocardial neutral lipid content in type 2 diabetic patients (Rodriguez-Calvo et al., 2019), thus proposing this molecule as an emerging biomarker for ectopic fat deposition in non-adipose tissues. Specifically in liver, serum FABP4 has been associated with NAFLD in both apparently healthy subjects (Kim et al., 2011) and type 2 diabetic patients (Koh et al., 2009). Additionally, experimental studies have shown that exogenous FABP4 induces intracellular lipid accumulation in HepG2 liver cells, thereby suggesting an active role of FABP4 in the liver fat deposition (Bosquet et al., 2016). Nevertheless, the relationship of serum FABP4 and FLI has been only explored in non-metabolic subjects (Jeon et al., 2013; Xu et al., 2019).

Therefore, the aim of this work was to analyze the relationship between circulating FABP4 and liver steatosis assessed by FLI in patients at increased cardiometabolic risk.

## Materials and Methods

### Subjects

A total of 389 individuals attending the Vascular Medicine and Metabolism Unit from the Saint Joan University Hospital (Reus, Spain) due to lipid metabolism disturbances and diabetes, overweight/obesity, or metabolic syndrome and willing to participate in the study were sequentially recruited. Anamnesis, anthropometric, and physical examination was performed using standardized procedures at the time of clinical visits. Individuals taking lipid-lowering medications underwent a wash-out period of at least 6 weeks (8 weeks if they were on fibrates) to avoid bias. Subjects with known chronic lung, renal, or chronic liver disease, cancer, or any other serious disease were excluded (Girona et al., 2019). Individuals with an alcohol consumption above 20 g/day (females) or 40 g/day (males) were not included. Viral or autoimmune hepatic diseases were excluded when clinically indicated. None of the included patients had clinical evidence of viral or autoimmune liver disease. Diabetes (*N* = 264), obesity (*N* = 202), and metabolic syndrome (*N* = 291) were diagnosed according to standard clinical criteria. Specifically, the presence of diabetes was defined as fasting concentration of blood sugar ≥126 mg/dl, glycated hemoglobin A1c > 6.5, glucose after a 2 h Oral Tolerance Tests ≥200 mg/dl or being treated with antidiabetic drugs, according to international criteria (American Diabetes Association, 2019), obesity as body mass index (BMI) ≥ 30 Kg/m^2^, and the presence of metabolic syndrome was defined according to the Adult Treatment Panel III criteria (National Cholesterol Education Program [NCEP] Expert Panel on Detection, Evaluation, and Treatment of High Blood Cholesterol in Adults, 2002). Arterial hypertension was defined as systolic and diastolic blood pressures ≥140 mm Hg and ≥90 mm Hg, respectively. Liver steatosis was defined as FLI ≥ 60. Written informed consent was obtained from all participants. The study was approved by Hospital Ethics Committee (Pere Virgili Health Research Institute; ethical approval code: 200/2018) and was performed in full compliance with the Declaration of Helsinki.

### Clinical and Laboratory Determinations

Demographic and clinical characteristics including age, gender, both systolic and diastolic blood pressure, weight and waist circumference were obtained at the point of study inclusion. BMI was calculated as follows = weight (kg)/height (m)^2^.

Venous blood sample were obtained from each patient in the study cohort after overnight fasting. Fasting blood was immediately centrifuged at 1500 *g* for 15 min at 4°C, and serum samples were aliquoted and stored at −80°C in the BioBank at our center until analysis. Biochemical parameters [glucose, glycated hemoglobin A1c (HbA1c), ultra-sensitive C-reactive protein (usCRP), creatinin], lipids [total cholesterol, triglycerides and high-density lipoprotein cholesterol (HDLc)], apolipoproteins [apolipoprotein A1 (apoA1) and apolipoprotein B100 (apoB100)], transaminases [aspartate aminotransferase (AST) and alanine aminotransferase (ALT)] and gamma-glutamyl transpeptidase (GGT) were measured using colorimetric, enzymatic and immunoturbidimetric assays, respectively (Spinreact, SA, Spain; Wako Chemicals GmbH, Germany; and Polymedco, New York, NY, United States; CV < 4%), which were adapted to the Cobas Mira Plus Autoanalyser (Roche Diagnostics, Spain). The low-density lipoprotein cholesterol (LDL-C) levels were calculated with the use of the Friedewald equation: LDL-C = total cholesterol − (HDL-C + [triglycerides ÷ 5]).

### Fatty Liver Index Determination

FLI was calculated using an algorithm based on BMI, waist circumference, triglycerides and GGT, which shows an accuracy of 0.84 (95% CI 0.81–0.87) detecting fatty liver. Specifically, FLI was calculated according to the following formula: FLI = (e^0.953 × loge (triglycerides) + 0.139 × BMI + 0.718 × loge (GGT) + 0.053 × waist circumference − 15.745^) / (1 + e^0.953 × loge (triglycerides) + 0.139 × BMI + 0.718 × loge (GGT) + 0.053 × waist circumference − 15.745^) × 100. Liver steatosis was defined as a FLI ≥ 60, as previously described (Bedogni et al., 2006).

### Serum Fatty Acid Binding Protein 4 Determination

The serum FABP4 levels were assessed using a commercial sandwich enzyme-linked immunosorbent assay kits (Biovendor, Brno, Czechia) with intra- and inter-assay coefficients of variation estimated at 5%. Briefly, serum samples were 10-fold diluted in the sample dilution buffer and then, the diluted samples, the standards and the quality controls were incubated with in duplicates in microplate wells pre-coated with polyclonal anti-human FABP4 antibody for 60 min on an orbital shaker. After washing with the wash solution, wells were incubated with the biotin labeled polyclonal anti-human FABP4 antibody for 60 min, washed and incubated with streptavidin-horseradish peroxidase (HRP) conjugate for 30 min. After a last washing, the substrate solution was added and the microtiter plate were incubated for 10 min protected from the direct sunlight. Then reaction was stopped by addition of acidic solution and absorbance of the resulting product was measured at 450 nm. The serum FABP4 concentrations were determined using a standard curve constructed with the kit’s standards.

### Statistical Analysis

The normality of continuous variables was determined by the Kolmogorov–Smirnov test. Continuous variables are expressed as median and interquartile range, unless otherwise indicated. Categorical variables are expressed as numbers with percentages. Differences in the FLI and FABP4 between patients with diabetes, obesity and metabolic syndrome were analyzed by the Mann–Whitney test. Correlations between two variables were performed by using the Spearman’s Rho coefficient (ρ). Univariate and multivariate linear regression models were constructed to search for independent relationships between FLI (dependent variable) and serum FABP4. Data are expressed as standardized beta (β). The study population was stratified in those with FLI ≥ 60 and those with FLI < 60 (Bedogni et al., 2006). Univariate and multivariate logistic binary regression models were performed for dichotomous variables to assess the risk of liver steatosis based on the serum FABP4 levels. The results are presented as odds ratios (OR) and 95% confidence interval (CI). Statistical analyses were performed using SPSS software (IBM SPSS Statistics, version 22.0). Differences were considered statistically significant at *P* < 0.05.

## Results

### Characteristics of the Study Population

The clinical, anthropometric and biochemical characteristics of the study population are showed in Supplementary Table 1. Out of 389 subjects included, 191 were men and 197 women. The median age of the study population was 62.0 (25.0–85.0) years. 17.2% of the subjects were usual smokers and 20.8% ex-smokers. A total of 49.1% had a clinical history of arterial hypertension. Diabetes, obesity, and metabolic syndrome were present in 67.9, 51.9, and 74.8%, respectively. A total of 63.8% of the patients showed liver steatosis, with a median FLI of 76.6 (42.7–94.3). The median serum FABP4 levels were 25.3 (16.7–38.3) ng/mL.

Obese individuals showed higher weight, waist circumference and BMI than diabetic and metabolic syndrome individuals. Additionally, obese patients showed higher FLI than the other study groups. Diabetic patients had higher HbA1c and lower triglycerides than the metabolic syndrome individuals. No significant differences were found among the other studied variables in the three study groups (Supplementary Table 2).

### Serum Fatty Acid Binding Protein 4 Levels Are Associated With Liver Injury and Inflammation Hallmarks in Metabolic Patients

We explored the potential correlations between the serum levels of FABP4 and hallmarks of liver injury, including AST, ALT, and GGT (Supplementary Table 3). No significant correlations were found between serum FABP4 and any transaminase, neither in the whole population nor in metabolic patients. Nevertheless, serum FABP4 was found positively correlated with GGT in all studied groups after adjusting for age, gender, glucose, triglycerides, apoA1, and apoB100. Additionally, serum FABP4 was strongly associated with the inflammatory hallmark usCRP in both the whole population and the metabolic patients (*P* < 0.001 for all comparisons) (Supplementary Table 3).

### Serum Fatty Acid Binding Protein 4 Levels Are Associated With the Fatty Liver Index in Metabolic Patients

**FIGURE 1:**
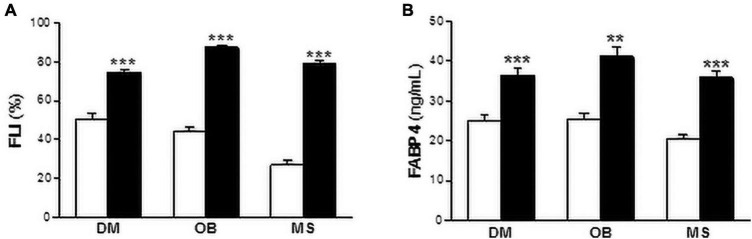
Both FLI and serum FABP4 levels are increased in metabolic patients. FLI **(A)** and serum FABP4 levels **(B)** in diabetic (DM), obese (OB) and metabolic syndrome (MS) patients. Black bars correspond to metabolic patients and white bars to non-metabolic individuals. Data are expressed as the means ± SEM. **P < 0.01, ***P < 0.001 vs control non-metabolic patients.

We next investigated whether FABP4 was associated with liver fat content assessed using the FLI. As it is shown in Figure 1, FLI was increased in diabetic (non-DM: 50.27 ± 3.10%; DM: 74.61 ± 1.50%, *P* < 0.001), obese (non-OB: 44.58 ± 2.01%; OB: 87.36 ± 0.95%, *P* < 0.001) and metabolic syndrome patients (non-MS: 26.97 ± 2.38%; MS: 79.15 ± 1.20%, *P* < 0.001) compared with subjects without the abovementioned metabolic disturbances (Figure 1A). Similarly, serum FABP4 levels were increased in patients with diabetes (non-DM: 25.20 ± 1.26 ng/mL; DM: 36.39 ± 1.82 ng/mL, *P* < 0.001), obesity (non-OB: 25.51 ± 1.40 ng/mL; OB: 41.24 ± 2.32 ng/mL, *P* < 0.01) and metabolic syndrome (non-MS: 20.39 ± 1.09 ng/mL; MS: 35.90 ± 1.59 ng/mL, *P* < 0.001) (Figure 1B).

Serum FABP4 levels positively correlated to FLI in the whole population (ρ = 0.387, *P* < 0.001), diabetic (ρ = 0.444, *P* < 0.001), obese (ρ = 0.392, *P* < 0.001), and metabolic syndrome (ρ = 0.300, *P* < 0.001) patients. After adjusting for age and gender (^*^), or for age, gender, glucose, triglycerides, apoA1, apoB100, and AST (^#^), the relationships remained statistically significant (*P* < 0.001 for all comparisons) (Table 1). Additionally, multivariate linear regression models performed with FLI designated as the dependent variable and with FABP4, age and gender (Model 1), or with FABP4, age, gender, glucose, triglycerides, apoA1, apoB100, and AST (Model 2), showed the independent relationships between FLI and serum FABP4 levels in metabolic subjects (Table 2). In the Model 1, FLI was independently associated with serum FABP4 in the whole population (*R*^2^ = 0.127, standardized β = 0.323, *P* < 0.001), as well as in diabetic (*R*^2^ = 0.165, standardized β = 0.267, *P* < 0.001), obese (*R*^2^ = 0.149, standardized β = 0.303, *P* < 0.001) and metabolic syndrome (*R*^2^ = 0.170, standardized β = 0.259, *P* < 0.001) patients. The positive relationships between serum FLI and serum FABP4 were improved in the Model 2 (All: *R*^2^ = 0.424, standardized β = 0.255, *P* < 0.001; DM: *R*^2^ = 0.306, standardized β = 0.251, *P* < 0.001; OB: *R*^2^ = 0.290, standardized β = 0.308, *P* = 0.001; MS: *R*^2^ = 0.286, standardized β = 0.256, *P* < 0.001).

**TABLE 1:** Relationships between serum FABP4 and FLI.

**Group**	**All**		**Diabetes**		**Obesity**		**Metabolic syndrome**	
	ρ	***P***	ρ	***P***	ρ	***P***	ρ	***P***
Unadjusted	0.387	<0.001	0.444	<0.001	0.392	<0.001	0.300	<0.001
Adjusted*	0.318	<0.001	0.273	<0.001	0.298	<0.001	0.265	<0.001
Adjusted^#^	0.305	<0.001	0.263	<0.001	0.313	<0.001	0.282	<0.001

Spearman correlations. Significance (*P*-values) of rho coefficients (ρ) between serum FABP4 and FLI are reported in the whole population (all), diabetes, obesity, and metabolic syndrome patients (unadjusted). *P*-values corrected by age, gender (*) and by age, gender, glucose, triglycerides, apoA1, apoB100, and AST (^#^).

**TABLE 2:** Associations between serum FABP4 and FLI.

**Group**	**All**			**Diabetes**			**Obesity**			**Metabolic syndrome**		
	β	***P***	***R*** ^2^	β	***P***	***R*** ^2^	β	***P***	***R*** ^2^	β	***P***	***R*** ^2^
Crude	0.276	<0.001	0.076	0.267	<0.001	0.071	0.259	<0.001	0.067	0.193	0.001	0.037
Model 1	0.323	<0.001	0.127	0.267	<0.001	0.165	0.303	<0.001	0.149	0.259	<0.001	0.170
Model 2	0.255	<0.001	0.424	0.251	<0.001	0.306	0.308	<0.001	0.290	0.256	<0.001	0.286

*Univariate (Crude model) and multivariate stepwise linear regression analysis showing associations between serum FABP4 and FLI in the whole population (All), diabetes, obesity and metabolic syndrome patients. FLI was entered as a dependent variable and FABP4, age, gender (Model 1) and FABP4, age, gender, glucose, triglycerides, apoA1, apoB100 and AST (Model 2) were subsequently entered as independent variables. Data are expressed as standardized beta (β) and *P*-values and *R*^2^ are shown.*

### Serum Fatty Acid Binding Protein 4 Levels Are Indicators for the Likelihood of Liver Steatosis in Metabolic Patients

**FIGURE 2:**
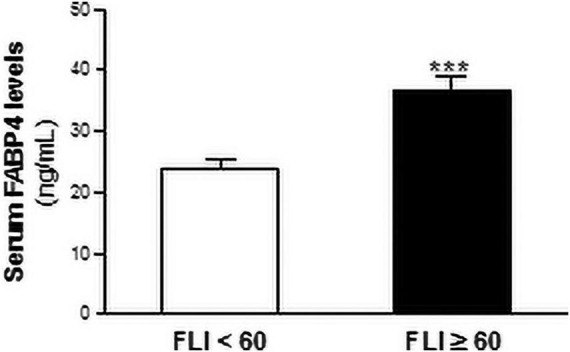
Serum FABP4 levels are increased in patients with liver steatosis. Serum FABP4 levels are shown in patients with FLI ≥ 60 (black bars, *N* = 247) or those with FLI < 60 (white bars, N = 140). Data are expressed as the means ± SEM. ***P < 0.001 vs FLI < 60 individuals.

Next, the population were stratified in those with FLI ≥ 60 and those with FLI < 60, according to the Bedogni criteria (Bedogni et al., 2006). The serum FABP4 levels were significantly increased in patients with FLI ≥ 60 (36.86 ± 2.13 ng/mL) compared with those with FLI < 60 (23.88 ± 1.17 ng/mL) (~ 1.5-fold, *P* < 0.01; vs FLI < 60) (Figure 2).

Then, univariate and multivariate logistic binary regression models were performed to assess the risk of liver steatosis (i.e., FLI ≥ 60) based on the serum FABP4 levels (Table 3). Serum FABP4 was positively related with liver steatosis in the whole population, in diabetic and metabolic syndrome, but not in obese patients in the crude model. After adjustment for age and gender (Model 1) and for age, gender, glucose, triglycerides, apoA1, apoB100, and AST (Model 2), the associations between FABP4 and liver steatosis were significant in all the studied groups, including obese patients.

**TABLE 3:** Crude and adjusted models used to assess the association between the serum FABP4 and liver steatosis.

**Group**	**All**		**Diabetes**		**Obesity**		**Metabolic syndrome**	
	**OR (95% CI)**	***P***	**OR (95% CI)**	***P***	**OR (95% CI)**	***P***	**OR (95% CI)**	***P***
Crude	1.04 (1.02–1.05)	<0.001	1.03 (1.01–1.05)	0.001	1.04 (1.00–1.09)	0.069	1.02 (1.00–1.03)	0.048
Model 1	1.06 (1.04–1.08)	<0.001	1.05 (1.03–1.08)	<0.001	1.08 (1.01–1.14)	0.016	1.05 (1.02–1.07)	<0.001
Model 2	1.04 (1.02–1.07)	<0.001	1.04 (1.02–1.07)	0.001	1.08 (1.00–1.17)	0.041	1.04 (1.01–1.06)	0.003

*Logistic regression models (odds ratio; OR and 95% confidence interval; CI). Model 1 was adjusted by age and gender, and Model 2 was adjusted by age, gender, glucose, triglycerides, apoA1, apoB100, and AST (METHOD = Enter).0.001 vs FLI < 60 individuals.*

## Discussion

The ectopic fat accumulation in liver is considered as the main precursor for NAFLD (Bonora and Targher, 2012; Wong et al., 2015; Kanwal et al., 2016). Nevertheless, its diagnosis remains a difficult task (Friedman et al., 2018). In this work, we explore the potential associations between serum FABP4 and liver steatosis assessed by FLI in individuals at increased cardiometabolic risk.

FLI is an algorithm widely used in epidemiologic studies as surrogate indicator of liver fat content (Koehler et al., 2013; Meffert et al., 2014). Nevertheless, the associations between serum FABP4 and FLI have been only explored in non-metabolic individuals (Jeon et al., 2013; Xu et al., 2019). Our study population is composed by metabolic patients, including diabetic, obese, and metabolic syndrome patients. Interestingly, both serum FABP4 levels and FLI were higher in our three groups of metabolic patients, compared to those individuals that no shown the abovementioned metabolic alterations. Serum FABP4 positively correlated with FLI in the whole population as well as in diabetic, obese, and metabolic syndrome patients. Additionally, multiple regression analysis showed the independent relationships between FLI and serum FABP4 levels in metabolic patients. Next, we explored the role of serum FABP4 as independent indicator of liver steatosis. First, we stratified our population in those with FLI ≥ 60 and those with FLI < 60, and liver steatosis was defined as FLI ≥ 60 according to the Bedogni criteria (Bedogni et al., 2006). In line with our previous data, the serum FABP4 levels were increased in patients with liver steatosis. Moreover, multivariate logistic regression models showed that serum FABP4 was independently associated with likelihood for liver steatosis in metabolic patients. Therefore, our data point out that FABP4 is strongly related to FLI in all the studies groups. The similar association between FABP4 an FLI in the different populations might be due to the fact that metabolic patients share common characteristics, such as insulin resistance. Nevertheless, in an analysis performed in a subset of the participants including HbA1c as a subrogate indicator of insulin resistance, the relationships between FABP4 and FLI remained statistically significant, thereby indicating that the similar relationship between these two variables in the different populations may go beyond its common characteristics.

Our data are in line with those reported in non-metabolic individuals. Xu et al. (2019) have recently reported that serum FABP4 is a positive determinant of FLI in normal glucose tolerance individuals. Moreover, in a prospective study performed in healthy individuals, Jeon et al. (2013) identified that serum FABP4 levels were found associate with FLI as a predictive indicator of NAFLD. Since liver steatosis is still a reversible step in the NAFLD progression, serum FABP4 may contribute to identify metabolic patients in the early stages of NAFLD, which would lead to initiate preventive therapies before the disease progression to more advanced irreversible stages, such as NASH or hepatocellular carcinoma. The use of FABP4 as an indicator of liver steatosis would be a diagnostic advantage over image-based methods, since this well-established non-invasive method would reduce patient discomfort and improve cost-effectiveness. Furthermore, serum FABP4 determination would not only improve the detection of the initial phases of the NAFLD, but also would allow the monitoring of the disease progression.

Additionally, we explored the potential relationship between serum FABP4 and hallmarks of liver injury. Despite no significant correlations were found between serum FABP4 and transaminases, it is worth to note that these molecules may not be reliable markers for the disease, since 75% of patients with NAFLD do not show elevated transaminases levels (Clark et al., 2003). Nevertheless, serum FABP4 was positively correlated with GGT and with usCRP, thereby suggesting that serum FABP4 is related to the liver injury and inflammation, which are characteristics of the advanced stages of the NAFLD. Actually, both GGT and CRP were increased in NASH patients (Cao et al., 2013), and serum GGT was independently associated to the presence of NASH (Leite et al., 2011). Therefore, although further studies are necessary to fully explore this hypothesis, serum FABP4 may not only reflect liver steatosis, but also NASH.

Our study has some limitations. First, the cross-sectional design precludes stablishing causal relationships between serum FABP4 and liver steatosis. Nevertheless, the prospective study performed by Jeon et al. (2013) strongly supports this conclusion. Additionally, experimental studies have shown an increased in the intracellular lipid content in a liver cell line stimulated with exogenous FABP4 (Bosquet et al., 2016), thus suggesting a cause-effect relationship between circulating FABP4 and the ectopic fat deposition in liver. Fatty liver was assessed by FLI which is a subrogated of imaging techniques (Cuthbertson et al., 2014); however, further validation by imaging techniques and/or liver biopsy is warranted before to fully consider serum FABP4 as a realistic option for liver steatosis determination.

## Conclusion

Overall, our findings strongly support that circulating FABP4 is related to the ectopic lipid content in the liver, thereby proposing this molecule as a potential non-invasive tool for the diagnosis of the early stages of NAFLD in individuals at increase cardiometabolic risk.

## Data Availability Statement

The original contributions presented in the study are included in the article/Supplementary Material, further inquiries can be directed to the corresponding authors.

## Ethics Statement

The studies involving human participants were reviewed and approved by the Pere Virgili Health Research Institute; ethical approval code: 200/2018. The patients/participants provided their written informed consent to participate in this study.

## Author Contributions

RR-C and LM: conceptualization. DI and NP: data curation. RR-C, JM-V, JG, and NM-M: formal analysis. RR-C: writing – original draft preparation, visualization, and supervision. RR-C, JM-V, JG, DI, NM-M, JM, NP, and LM: writing – review and editing. JG and LM: funding acquisition. All authors contributed to the article and approved the submitted version.

## Conflict of Interest

The authors declare that the research was conducted in the absence of any commercial or financial relationships that could be construed as a potential conflict of interest.

## Publisher’s Note

All claims expressed in this article are solely those of the authors and do not necessarily represent those of their affiliated organizations, or those of the publisher, the editors and the reviewers. Any product that may be evaluated in this article, or claim that may be made by its manufacturer, is not guaranteed or endorsed by the publisher.
