# Supplementary material for: Relationship Between Fatty Acid Binding Protein 4 and Liver Fat in Individuals at Increased Cardiometabolic Risk
Source: Front Physiol. 2021 Dec 13;12:781789. doi: 10.3389/fphys.2021.781789 (PMC8711782; doi:10.3389/fphys.2021.781789)
Supplement: Supplementary file 1 [file Data_Sheet_1.docx]

**Supplementary Table 1.** Clinical and biochemical characteristics of the study subjects.

|  | **N=389** | |
| --- | --- | --- |
| ***Clinical data*** |  | |
| Age (years) | 62.0 (25.0-85.0) | |
| Gender (M/F)^a^ | 191/197 | |
| Smokers^a^ | 67 (17.2%) | |
| Ex-smokers^a^ | 81 (20.8%) | |
| Hypertension^b^ | 191 (49.1%) | |
| Diabetes | 264 (67.9%) | |
| Obesity | 202 (51.9%) | |
| Metabolic syndrome | 291 (74.8%) | |
| Steatosis | 248(63.8%) | |
| ***Analytical data*** | |  |
| Systolic BP (mmHg)^d^ | 135.0 (125.0-149.0) | |
| Diastolic BP (mmHg)^d^ | 80.0 (72.0-85.0) | |
| Weight (Kg) | 83.0 (72.0-94.1) | |
| Waist circumference (cm) | 102.0 (94.5-112.9) | |
| BMI (Kg/m^2^) | 30.0 (27.2-34.9) | |
| FLI (%) | 76.6 (42.7-94.3) | |
| Glucose (mg/dL) | 126.0 (101.0-164.0) | |
| HbA1c (%)^e^ | 6.4 (5.7-7.5) | |
| Triglycerides (mmol/L) | 1.6 (1.0-2.6) | |
| Cholesterol (mmol/L) | 5.3 (4.6-6.4) | |
| HDLc (mmol/L) | 1.4 (1.2-1.6) | |
| apoA1 (mg/dL) | 136.0 (128.0-147.0) | |
| LDLc (mmol/L) | 3.3 (2.6-4.1) | |
| apoB100 (mg/dL) | 104.0 (85.5-124.0) | |
| usCRP (mg/L)^f^ | 2.1 (1.1-3.6) | |
| AST (U/L) | 23.0 (20.0-28.0) | |
| ALT (U/L) | 17.0 (12.0-25.0) | |
| GGT (U/L) | 24.0 (17.0-40.0) | |
| Creatinine (µmol/L)^g^ | 46.0 (36.2-59.2) | |
| FABP4 (ng/mL)^h^ | 25.3 (16.7-38.3) | |

^a^N=388; ^b^N=355; ^c^N=342; ^d^N=341; ^e^N=300; ^f^N=385; ^g^N=276; ^h^N=387

Data are shown as n (percentage) or median (interquartile range).

**Supplementary Table 2.** Biochemical characteristics in the three study groups.

|  |  | **Diabetes**  (N=264) |  | **Obesity**  (N=202) |  | **Metabolic syndrome**  (N=291) |
| --- | --- | --- | --- | --- | --- | --- |
| Systolic BP (mmHg)^a^ |  | 139.0 (130.0-152.0) |  | 140.0 (130.0-153.0) |  | 139.0 (130.0-150.3) |
| Diastolic BP (mmHg)^a^ |  | 80.0 (75.0-86.0) |  | 81.0 (76.0-90.0) |  | 80.0 (75.0-88.0) |
| Weight (Kg) |  | 85.0 (73.0-98.2) |  | 92.9 (84.1-104.2)*** |  | 86.0 (76.6-98.7) |
| Waist circumference (cm) |  | 104.0 (99.0-116.0) |  | 112.0 (104.0-122.0)*** |  | 106.0 (100.0-116.0) |
| BMI (Kg/m^2^) |  | 31.0 (28.5-36.4) |  | 34.7 (31.8-38.7)*** |  | 32.1 (29.2-36.5) |
| FLI (%) |  | 82.9 (58.0-96.0) |  | 93.3 (81.8-97.5)*** |  | 85.7 (65.6-96.2) |
| Glucose (mg/dL) |  | 145.0 (119.0-178.9) |  | 137.9 (112.8-174.2) |  | 138.0 (112.0-173.0) |
| HbA1c (%)^b^ |  | 6.7 (6.0-7.7)^#^ |  | 6.3 (5.6-7.6) |  | 6.4 (5.7-7.5) |
| Triglycerides (mmol/L) |  | 1.7 (1.2-2.9) ^#^ |  | 2.0 (1.3-3.1) |  | 2.0 (1.3-3.3) |
| Cholesterol (mmol/L) |  | 5.4 (4.6-6.5) |  | 5.5 (4.7-6.7) |  | 5.6 (4.7-6.6) |
| HDLc (mmol/L) |  | 1.4 (1.2-1.6) |  | 1.4 (1.2-1.5) |  | 1.4 (1.2-1.5) |
| apoA1 (mg/dL) |  | 138.0 (129.0-148.0) |  | 134.0 (126.8-147.0) |  | 134.0 (127.0-145.0) |
| LDLc (mmol/L) |  | 3.3 (2.6-4.3) |  | 3.5 (2.7-4.5) |  | 3.5 (2.7-4.3) |
| apoB100 (mg/dL) |  | 105.0 (85.3-127.0) |  | 110.5 (90.0-131.3) |  | 109.0 (90.0-129.0) |
| usCRP (mg/L)^c^ |  | 2.4 (1.5-3.9) |  | 2.8 (1.5-4.5) |  | 2.4 (1.4-3.8) |
| AST (U/L) |  | 23.0 (19.0-28.8) |  | 24.0 (20.0-31.0) |  | 23.0 (20.0-30.0) |
| ALT (U/L) |  | 18.0 (12.0-26.0) |  | 20.5 (14.0-30.0) |  | 19.0 (13.0-28.0) |
| GGT (U/L) |  | 26.5 (18.0-43.0) |  | 28.0 (19.8-46.0) |  | 27.0 (18.0-43.0) |
| Creatinine (µmol/L)^d^ |  | 42.4 (34.5-54.8) |  | 42.4 (34.5-54.8) |  | 43.3 (34.5-54.8) |
| FABP4 (ng/mL)^e^ |  | 27.6 (18.4-41.6) |  | 32.2 (20.5-46.8) |  | 28.3 (19.0-41.6) |

Data are shown as n (percentage) or median (interquartile range). ^a^Diabetes: N=217; Obese: N=174; Metabolic syndrome: N=257. ^b^Diabetes: N=252; Obese: N=176; Metabolic syndrome: N=259. ^c^Diabetes: N=261; Obese: N=201; Metabolic syndrome: N=290. ^d^Diabetes: N=181; Obese: N=134; Metabolic syndrome: N=190. ^e^Diabetes: N=262; Obese: N=202; Metabolic syndrome: N=290. *^***^P*<0.001 vs Diabetes and Metabolic Syndrome; *^#^P*<0.05 vs Metabolic Syndrome

**Supplementary Table 3.** Relationships between serum FABP4 and liver injury hallmarks.

| **Group** |  | **All** | | |  | **Diabetes** | | |  | **Obesity** | | |  | **Metabolic syndrome** | | |
| --- | --- | --- | --- | --- | --- | --- | --- | --- | --- | --- | --- | --- | --- | --- | --- | --- |
| **AST** |  | ρ |  | *P* |  | ρ |  | *P* |  | ρ |  | *P* |  | ρ |  | *P* |
| **Unadjusted** |  | -0.051 |  | 0.318 |  | -0.035 |  | 0.577 |  | -0.091 |  | 0.197 |  | -0.088 |  | 0.135 |
| **Adjusted*** |  | 0.068 |  | 0.182 |  | 0.042 |  | 0.502 |  | 0.036 |  | 0.616 |  | 0.038 |  | 0.522 |
| **Adjusted^#^** |  | 0.054 |  | 0.297 |  | 0.043 |  | 0.493 |  | 0.036 |  | 0.613 |  | 0.042 |  | 0.483 |
| **ALT** |  | ρ |  | *P* |  | ρ |  | *P* |  | ρ |  | *P* |  | ρ |  | *P* |
| **Unadjusted** |  | 0.059 |  | 0.247 |  | 0.074 |  | 0.231 |  | 0.009 |  | 0.901 |  | -0.025 |  | 0.668 |
| **Adjusted*** |  | 0.062 |  | 0.227 |  | 0.014 |  | 0.287 |  | 0.028 |  | 0.697 |  | -0.007 |  | 0.908 |
| **Adjusted^#^** |  | 0.035 |  | 0.501 |  | 0.003 |  | 0.958 |  | 0.026 |  | 0.719 |  | -0.004 |  | 0.948 |
| **GGT** |  | ρ |  | *P* |  | ρ |  | *P* |  | ρ |  | *P* |  | ρ |  | *P* |
| **Unadjusted** |  | 0.096 |  | 0.060 |  | 0.077 |  | 0.215 |  | -0.017 |  | 0.806 |  | -0.028 |  | 0.634 |
| **Adjusted*** |  | 0.152 |  | 0.003 |  | 0.134 |  | 0.031 |  | 0.116 |  | 0.104 |  | 0.137 |  | 0.020 |
| **Adjusted^#^** |  | 0.145 |  | 0.005 |  | 0.158 |  | 0.012 |  | 0.146 |  | 0.042 |  | 0.151 |  | 0.011 |
| **usCRP** |  | ρ |  | *P* |  | ρ |  | *P* |  | ρ |  | *P* |  | ρ |  | *P* |
| **Unadjusted** |  | 0.331 |  | <0.001 |  | 0.283 |  | < 0.001 |  | 0.330 |  | <0.001 |  | 0.255 |  | < 0.001 |
| **Adjusted*** |  | 0.317 |  | <0.001 |  | 0.265 |  | < 0.001 |  | 0.306 |  | <0.001 |  | 0.275 |  | < 0.001 |
| **Adjusted^#^** |  | 0.288 |  | <0.001 |  | 0.259 |  | < 0.001 |  | 0.295 |  | <0.001 |  | 0.268 |  | < 0.001 |

Spearman correlations. Significance (*P*-values) of rho coefficients (ρ) between serum FABP4 and AST, ALT, GGT and usCRP are reported in the whole population (All), Diabetes, Obesity and Metabolic syndrome patients (Unadjusted). *P* values corrected by age, gender (*) and by age, gender, glucose, triglycerides, apoA1 and apoB100 (^#^).
